# Supplementary material for: A Contribution to the Solid State Forms of Bis(demethoxy)curcumin: Co-Crystal Screening and Characterization
Source: Molecules. 2021 Jan 30;26(3):720. doi: 10.3390/molecules26030720 (PMC7866521; doi:10.3390/molecules26030720)
Supplement: Supplementary file 1 [file molecules-26-00720-s001.pdf]

Supplementary Material

S1. Characterization of BDMC, PHLO, and co-crystallization products of BDMC and PHLO, (a) DSC thermograms at a heating rate of 10°C·min-1, and 2°C min-1 (blue line): DSC curves of pure substances are shown as dotted lines, grey bar indicates eutectic melting; (b) PXRD diffractograms: characteristic peaks of pure PHLO and BDMC are marked as dotted green and orange lines, a PXRD diffractogram of a sample produced from eutectic melt was not measured due to decomposition.

Abbreviations: RSR-rapid solvent removal, SSE-slow solvent evaporation, LAG-liquid assisted grinding.

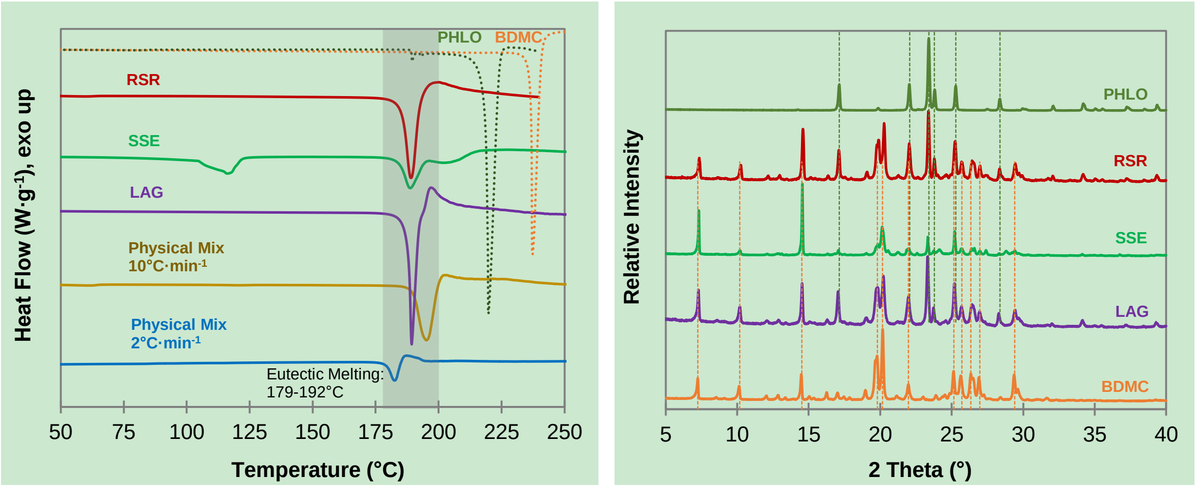

(a) (b)

S2. Thermogravimetric analysis (TGA-DSC) of pure HYQ, heating rate: 2°C·min-1.

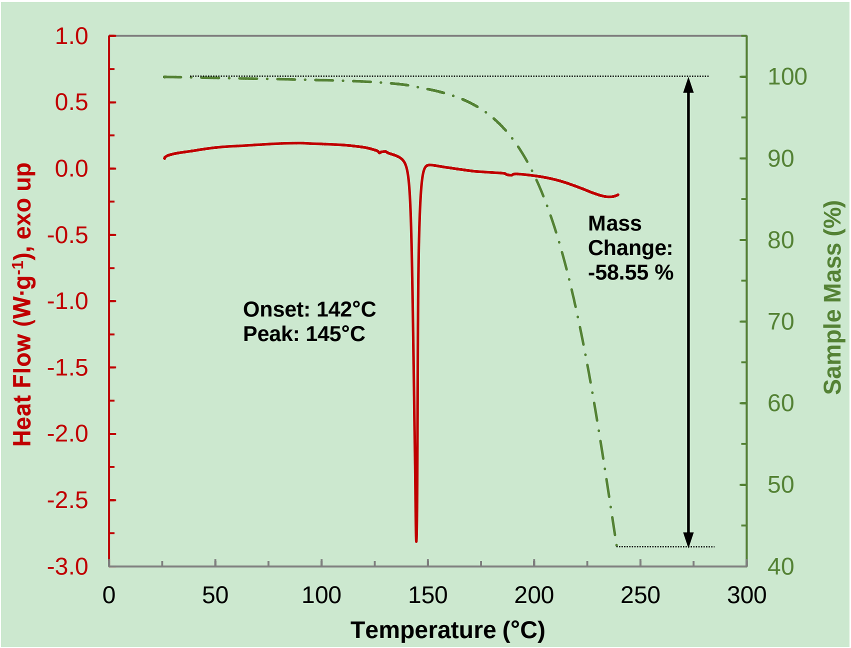

S3. Polarized light microscope images of HYQ-BDMC co-crystal grown from ethyl acetate, (a) BDMC crystallizes first from the solution, (b) starting nucleation of HYQ-BDMC co-crystals at a gas-liquid interface, (c) and (d) ongoing co-crystal nucleation, and growth as spherulites

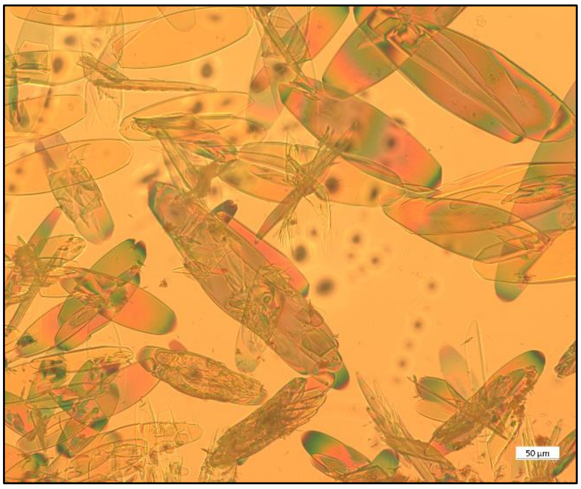

(a)

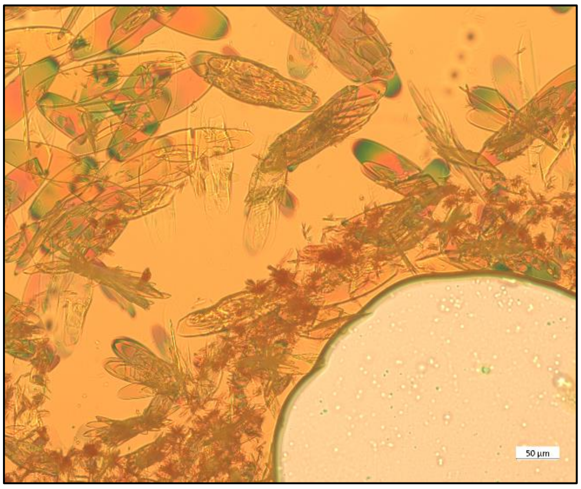

(b)

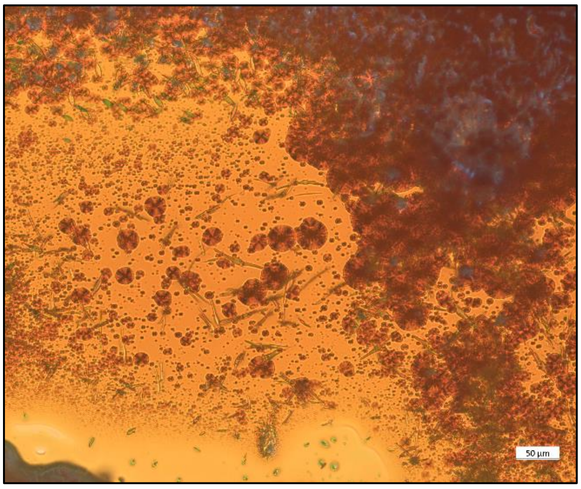

(c)

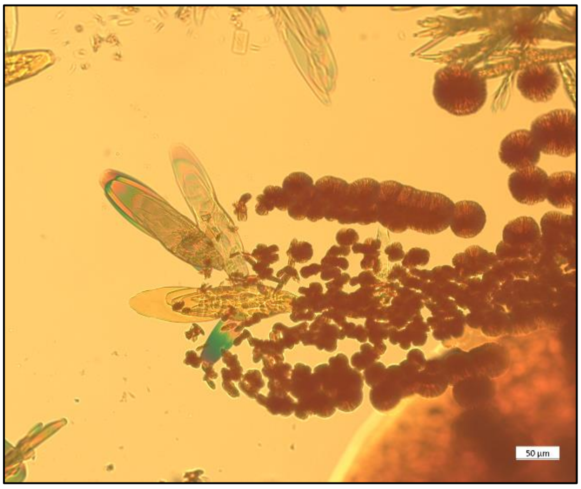

(d)

S4. SEM micrographs of (a) pure BDMC crystals at 200x magnification, (b) pure HYQ crystals at 250x magnification; detection of backscatter electrons

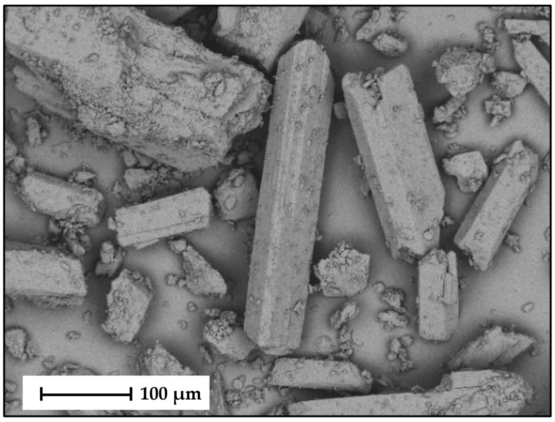

(a)

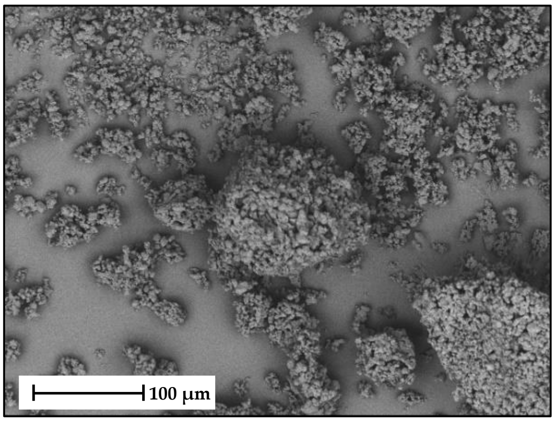

(b)
